# 2-[4-(2-Methyl­prop­yl)phen­yl]-*N*′-[(*E*)-1-phenyl­ethyl­idene]propane­hydrazide

**DOI:** 10.1107/S1600536808036817

**Published:** 2008-11-20

**Authors:** Hoong-Kun Fun, Samuel Robinson Jebas, K. V. Sujith, B. Kalluraya

**Affiliations:** a X-ray Crystallography Unit, School of Physics, Universiti Sains Malaysia, 11800 USM, Penang, Malaysia; b Department of Studies in Chemistry, Mangalore University, Mangalagangotri, Mangalore 574 199, India

## Abstract

In the title compound, C_21_H_26_N_2_O, the dihedral angle between the two aromatic rings is 85.90 (19)°. The propenone–hydrazide unit forms dihedral angles of 21.62 (8) and 72.83 (9)°, respectively, with the terminal and central aromatic rings. The 2-methyl­propyl group is disordered over two sites, with occupancies of 0.533 (13) and 0.467 (13). In crystal structure, mol­ecules are linked into centrosymmetric dimers by paired N—H⋯O and C—H⋯O hydrogen bonds. In addition, C—H⋯π inter­actions are observed.

## Related literature

For the pharmaceutical applications of ibuprofen, see: Palaska *et al.* (2002 ▶). For the synthesis of hydrazones, see: Rollas & Küçükgüzel (2007 ▶). For the pharmaceutical applications of hydrazones, see: Bedia *et al.* (2006 ▶); Rollas *et al.* (2002 ▶); Terzioglu & Gürsoy (2003 ▶). For a related structure, see: Fun *et al.* (2008 ▶). For bond-length data, see: Allen *et al.* (1987 ▶).
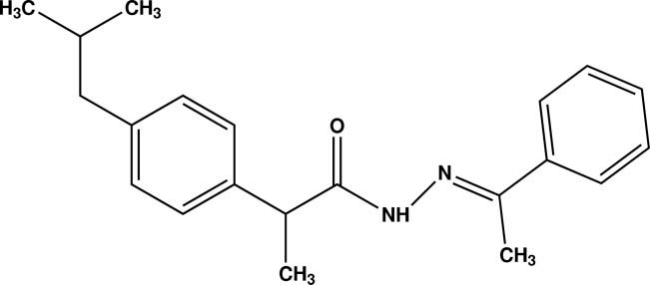


## Experimental

### 

#### Crystal data

C_21_H_26_N_2_O
*M* *_r_* = 322.44
Triclinic, 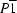

*a* = 5.4355 (2) Å
*b* = 10.2850 (4) Å
*c* = 17.3095 (6) Å
α = 80.821 (4)°
β = 84.312 (3)°
γ = 74.719 (3)°
*V* = 919.85 (6) Å^3^
*Z* = 2
Mo *K*α radiation
μ = 0.07 mm^−1^
*T* = 100.0 (1) K
0.22 × 0.20 × 0.15 mm

#### Data collection

Bruker APEXII CCD area-detector diffractometer
Absorption correction: multi-scan (*SADABS*; Bruker, 2005 ▶) *T*_min_ = 0.984, *T*_max_ = 0.989
12794 measured reflections
4238 independent reflections
2556 reflections with *I* > 2σ(*I*)
*R*_int_ = 0.063

#### Refinement

*R*[*F* ^2^ > 2σ(*F* ^2^)] = 0.072
*wR*(*F* ^2^) = 0.194
*S* = 1.07
4238 reflections
255 parameters
H-atom parameters constrained
Δρ_max_ = 0.33 e Å^−3^
Δρ_min_ = −0.26 e Å^−3^

### 

Data collection: *APEX2* (Bruker, 2005 ▶); cell refinement: *SAINT* (Bruker, 2005 ▶); data reduction: *SAINT*; program(s) used to solve structure: *SHELXTL* (Sheldrick, 2008 ▶); program(s) used to refine structure: *SHELXTL*; molecular graphics: *SHELXTL*; software used to prepare material for publication: *SHELXTL* and *PLATON* (Spek, 2003 ▶).

## Supplementary Material

Crystal structure: contains datablocks global, I. DOI: 10.1107/S1600536808036817/ci2710sup1.cif

Structure factors: contains datablocks I. DOI: 10.1107/S1600536808036817/ci2710Isup2.hkl

Additional supplementary materials:  crystallographic information; 3D view; checkCIF report

## Figures and Tables

**Table 1:** Hydrogen-bond geometry (Å, °) *Cg*1 is the centroid of the C1–C6 ring.

*D*—H⋯*A*	*D*—H	H⋯*A*	*D*⋯*A*	*D*—H⋯*A*
N2—H1*N*2⋯O1^i^	0.86 (2)	2.08 (2)	2.928 (3)	173 (2)
C20—H20*A*⋯O1^i^	0.96	2.31	3.247 (3)	165
C20—H20*B*⋯*Cg*1^ii^	0.96	2.75	3.609 (3)	150

Symmetry codes: (i) 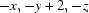; (ii) 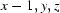.

**Fig. 1.**
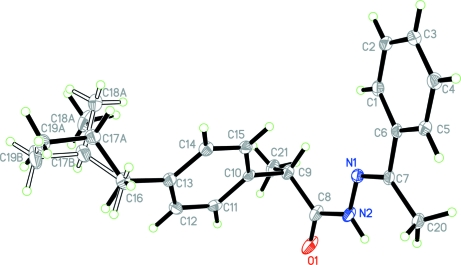
The molecular structure of the title compound, showing 50% probability displacement ellipsoids and the atom-numbering scheme. Both disorder components are shown.

**Fig. 2.**
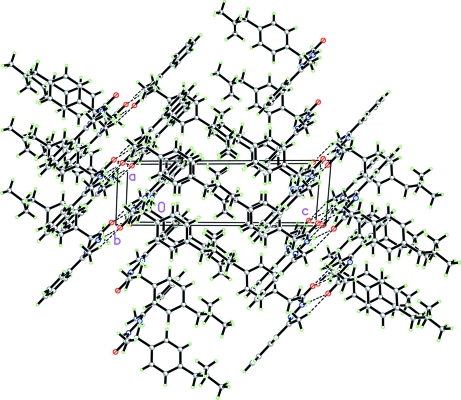
The crystal packing of the title compound viewed down the b axis. Only the major disorder component is shown.

## supplementary crystallographic information

### Comment

Ibuprofen belongs to the class of Non-Steroidal anti-Inflammatory Drugs (NSAIDs) with antipyretic, anti-inflammatory and analgesic properties (Palaska *et al.*, 2002). Hydrazones containing an azometine -NHN═CH- moiety are synthesized by heating the appropriate substituted hydrazines/hydrazides with aldehydes and ketones in solvents like ethanol, methanol, tetrahydrofuran, butanol, glacial acetic acid, ethanol-glacial acetic acid. Another synthetic route for the synthesis of hydrazones is the coupling of aryldiazonium salts with active hydrogen compounds (Rollas & Kuckguzel, 2007). Hydrazide-hydrazones compounds are not only intermediates but they are also very effective organic compounds of their own. Hydrazones have been demonstrated to possess antimicrobial, anticonvulsant, analgesic, anti-inflammatory, antiplatelet, antitubercular, anticancer and antitumoral activities (Bedia *et al.*, 2006; Rollas *et al.*, 2002; Terzioglu & Gursoy, 2003). Prompted by these and in continuation of our  work, (Fun *et al.*, 2008) we are interested in the synthesis and crystal structure determination of ibuprofen derivatives. We report here the crystal structure of the title compound (I).

Bond lengths in the title molecule (Fig.1) have normal values (Allen *et al.*, 1987). The two phenyl rings are essentially planar, with the maximum deviation from planarity being 0.003 (3)Å for atom C3 in the (C1-C6) ring and 0.012 (3)Å for atom C10 in the (C10-C15) ring. The two phenyl rings form a dihedral angle of 85.90 (11)°, indicating that they are almost orthogonal to each other. The propenone-hydrazide unit (O1/N1/N2/C8-C9) forms dihedral angles of 21.62 (8)° and 72.83 (9)° with (C1-C6) and (C10-C15) rings, respectively.

The crystal packing is consolidated by inter-molecular N—H···O and C—H···O hydrogen bonds together with C—H···π interactions (Table 1) involving the (C1-C6) ring (Centroid Cg1).

### Experimental

The title compound was obtained by refluxing 2-[4-(2-methylpropyl)phenyl]propanehydrazide (0.01 mol) and acetophenone (0.01 mol) in ethanol (30 ml) with 3 drops of concentrated sulfuric acid for 1 h. The excess ethanol was removed from the reaction mixture under reduced pressure. The solid product obtained was filtered, washed with ethanol and dried. Single crystals suitable for X-ray analysis were obtained by slow evaporation of an ethanol solution (yield 87%; m.p.380–381 K).

### Refinement

The 2-methylpropyl group is disordered over two orientations with refined occupancies of 0.533 (13):0.467 (13). H atoms were positioned geometrically (N-H=0.86Å and C-H=0.93-0.98Å) and refined using a riding model with, U_iso_(H)=1.2U_equ_(C,N) and 1.5U_equ_(C_methyl_). A rotating group model was used for the methyl groups.

### Crystal data

**Table:**

C_21_H_26_N_2_O	*Z* = 2
*M**_r_* = 322.44	*F*_000_ = 348
Triclinic, *P*1	*D*_x_ = 1.164 Mg m^−^^3^
Hall symbol: -P 1	Mo *K*α radiation λ = 0.71073 Å
*a* = 5.4355 (2) Å	Cell parameters from 2156 reflections
*b* = 10.2850 (4) Å	θ = 2.6–26.3º
*c* = 17.3095 (6) Å	µ = 0.07 mm^−^^1^
α = 80.821 (4)º	*T* = 100.0 (1) K
β = 84.312 (3)º	Block, colourless
γ = 74.719 (3)º	0.22 × 0.20 × 0.15 mm
*V* = 919.85 (6) Å^3^	

### Data collection

**Table:**

Bruker SMART APEXII CCD area-detector diffractometer	4238 independent reflections
Radiation source: fine-focus sealed tube	2556 reflections with *I* > 2σ(*I*)
Monochromator: graphite	*R*_int_ = 0.063
*T* = 100.0(1) K	θ_max_ = 27.5º
φ and ω scans	θ_min_ = 1.2º
Absorption correction: multi-scan(SADABS; Bruker, 2005)	*h* = −7→7
*T*_min_ = 0.984, *T*_max_ = 0.989	*k* = −13→13
12794 measured reflections	*l* = −22→22

### Refinement

**Table:**

Refinement on *F*^2^	Secondary atom site location: difference Fourier map
Least-squares matrix: full	Hydrogen site location: inferred from neighbouring sites
*R*[*F*^2^ > 2σ(*F*^2^)] = 0.072	H atoms treated by a mixture of independent and constrained refinement
*wR*(*F*^2^) = 0.194	*w* = 1/[σ^2^(*F*_o_^2^) + (0.0958*P*)^2^] where *P* = (*F*_o_^2^ + 2*F*_c_^2^)/3
*S* = 1.07	(Δ/σ)_max_ < 0.001
4238 reflections	Δρ_max_ = 0.33 e Å^−^^3^
255 parameters	Δρ_min_ = −0.25 e Å^−^^3^
Primary atom site location: structure-invariant direct methods	Extinction correction: none

### Special details

**Table:**

Experimental. The data was collected with the Oxford Cyrosystem Cobra low-temperature attachment.
Geometry. All esds (except the esd in the dihedral angle between two l.s. planes) are estimated using the full covariance matrix. The cell esds are taken into account individually in the estimation of esds in distances, angles and torsion angles; correlations between esds in cell parameters are only used when they are defined by crystal symmetry. An approximate (isotropic) treatment of cell esds is used for estimating esds involving l.s. planes.
Refinement. Refinement of F^2^ against ALL reflections. The weighted R-factor wR and goodness of fit S are based on F^2^, conventional R-factors R are based on F, with F set to zero for negative F^2^. The threshold expression of F^2^ > 2sigma(F^2^) is used only for calculating R-factors(gt) etc. and is not relevant to the choice of reflections for refinement. R-factors based on F^2^ are statistically about twice as large as those based on F, and R- factors based on ALL data will be even larger.

### Fractional atomic coordinates and isotropic or equivalent isotropic displacement parameters (Å^2^)

**Table:**

	*x*	*y*	*z*	*U*_iso_*/*U*_eq_	Occ. (<1)
O1	−0.0323 (4)	1.15692 (18)	0.02969 (11)	0.0501 (6)	
N1	0.4274 (4)	0.90562 (19)	0.13372 (11)	0.0262 (5)	
N2	0.2392 (4)	0.9662 (2)	0.08205 (12)	0.0313 (5)	
C1	0.8421 (4)	0.8048 (2)	0.22728 (13)	0.0279 (5)	
H1A	0.7995	0.8977	0.2095	0.033*	
C2	1.0316 (5)	0.7513 (3)	0.27947 (14)	0.0310 (6)	
H2A	1.1146	0.8087	0.2967	0.037*	
C3	1.0993 (5)	0.6133 (3)	0.30636 (14)	0.0315 (6)	
H3A	1.2282	0.5778	0.3411	0.038*	
C4	0.9730 (5)	0.5284 (3)	0.28104 (14)	0.0313 (6)	
H4A	1.0165	0.4356	0.2990	0.038*	
C5	0.7821 (4)	0.5819 (2)	0.22888 (14)	0.0274 (5)	
H5A	0.6985	0.5241	0.2123	0.033*	
C6	0.7130 (4)	0.7198 (2)	0.20080 (13)	0.0244 (5)	
C7	0.5099 (4)	0.7754 (2)	0.14382 (13)	0.0250 (5)	
C8	0.1345 (5)	1.1015 (2)	0.07640 (14)	0.0342 (6)	
C9	0.2172 (5)	1.1812 (2)	0.13175 (13)	0.0285 (6)	
H9A	0.3966	1.1389	0.1424	0.034*	
C10	0.0556 (4)	1.1707 (2)	0.20861 (13)	0.0236 (5)	
C11	−0.2016 (4)	1.2400 (2)	0.21324 (14)	0.0276 (6)	
H11A	−0.2775	1.2898	0.1682	0.033*	
C12	−0.3465 (5)	1.2360 (2)	0.28371 (15)	0.0314 (6)	
H12A	−0.5168	1.2851	0.2854	0.038*	
C13	−0.2420 (5)	1.1600 (2)	0.35198 (14)	0.0288 (6)	
C14	0.0141 (5)	1.0878 (2)	0.34642 (14)	0.0285 (5)	
H14A	0.0887	1.0349	0.3909	0.034*	
C15	0.1596 (5)	1.0932 (2)	0.27617 (13)	0.0261 (5)	
H15A	0.3298	1.0441	0.2743	0.031*	
C16	−0.3971 (5)	1.1569 (3)	0.42892 (15)	0.0398 (7)	
H16A	−0.3843	1.0634	0.4501	0.048*	0.467 (13)
H16B	−0.5731	1.1986	0.4190	0.048*	0.467 (13)
H16C	−0.3175	1.0769	0.4631	0.048*	0.533 (13)
H16D	−0.5637	1.1492	0.4198	0.048*	0.533 (13)
C17A	−0.3180 (19)	1.2210 (10)	0.4913 (4)	0.0316 (18)	0.467 (13)
H17A	−0.1511	1.1634	0.5068	0.038*	0.467 (13)
C18A	−0.281 (2)	1.3628 (10)	0.4643 (5)	0.053 (3)	0.467 (13)
H18A	−0.1524	1.3595	0.4218	0.079*	0.467 (13)
H18B	−0.2269	1.3958	0.5070	0.079*	0.467 (13)
H18C	−0.4387	1.4228	0.4470	0.079*	0.467 (13)
C19A	−0.4977 (19)	1.2181 (10)	0.5642 (6)	0.037 (2)	0.467 (13)
H19A	−0.4286	1.2466	0.6057	0.055*	0.467 (13)
H19B	−0.5165	1.1272	0.5801	0.055*	0.467 (13)
H19C	−0.6615	1.2786	0.5530	0.055*	0.467 (13)
C17B	−0.4221 (17)	1.2748 (9)	0.4729 (4)	0.0370 (17)	0.533 (13)
H17B	−0.5134	1.3567	0.4401	0.044*	0.533 (13)
C18B	−0.1698 (15)	1.2984 (10)	0.4884 (4)	0.041 (2)	0.533 (13)
H18D	−0.0717	1.3101	0.4397	0.061*	0.533 (13)
H18E	−0.0771	1.2215	0.5221	0.061*	0.533 (13)
H18F	−0.2002	1.3787	0.5133	0.061*	0.533 (13)
C19B	−0.586 (2)	1.2606 (11)	0.5497 (6)	0.053 (2)	0.533 (13)
H19D	−0.6109	1.3408	0.5743	0.080*	0.533 (13)
H19E	−0.4999	1.1825	0.5841	0.080*	0.533 (13)
H19F	−0.7481	1.2496	0.5388	0.080*	0.533 (13)
C20	0.4176 (5)	0.6775 (2)	0.10498 (14)	0.0311 (6)	
H20A	0.3320	0.7251	0.0589	0.047*	
H20B	0.3010	0.6385	0.1407	0.047*	
H20C	0.5607	0.6065	0.0906	0.047*	
C21	0.1932 (6)	1.3288 (2)	0.09447 (15)	0.0388 (7)	
H21A	0.2250	1.3800	0.1325	0.058*	
H21B	0.0239	1.3677	0.0769	0.058*	
H21C	0.3155	1.3314	0.0506	0.058*	
H1N2	0.185 (4)	0.923 (2)	0.0516 (14)	0.026 (6)*	

### Atomic displacement parameters (Å^2^)

**Table:**

	*U*^11^	*U*^22^	*U*^33^	*U*^12^	*U*^13^	*U*^23^
O1	0.0774 (15)	0.0287 (10)	0.0408 (11)	0.0079 (10)	−0.0309 (11)	−0.0144 (8)
N1	0.0268 (11)	0.0257 (11)	0.0250 (10)	−0.0033 (8)	−0.0014 (8)	−0.0063 (8)
N2	0.0422 (13)	0.0252 (11)	0.0264 (11)	−0.0018 (9)	−0.0092 (10)	−0.0103 (9)
C1	0.0273 (13)	0.0247 (12)	0.0312 (13)	−0.0046 (10)	0.0033 (11)	−0.0089 (10)
C2	0.0264 (13)	0.0376 (14)	0.0324 (14)	−0.0090 (11)	0.0025 (11)	−0.0160 (11)
C3	0.0247 (13)	0.0370 (15)	0.0321 (14)	−0.0027 (11)	−0.0013 (11)	−0.0114 (11)
C4	0.0317 (14)	0.0266 (13)	0.0330 (14)	−0.0015 (11)	−0.0030 (11)	−0.0051 (10)
C5	0.0282 (13)	0.0236 (12)	0.0320 (13)	−0.0059 (10)	−0.0017 (10)	−0.0094 (10)
C6	0.0226 (12)	0.0265 (12)	0.0239 (12)	−0.0046 (10)	0.0058 (10)	−0.0098 (10)
C7	0.0257 (12)	0.0260 (12)	0.0231 (12)	−0.0047 (10)	0.0036 (10)	−0.0089 (10)
C8	0.0502 (16)	0.0248 (13)	0.0259 (13)	−0.0028 (12)	−0.0074 (12)	−0.0061 (10)
C9	0.0358 (14)	0.0227 (12)	0.0272 (13)	−0.0048 (10)	−0.0030 (11)	−0.0073 (10)
C10	0.0286 (12)	0.0191 (11)	0.0266 (12)	−0.0077 (9)	−0.0053 (10)	−0.0089 (9)
C11	0.0311 (14)	0.0214 (12)	0.0320 (13)	−0.0055 (10)	−0.0123 (11)	−0.0041 (10)
C12	0.0222 (12)	0.0308 (13)	0.0430 (15)	−0.0050 (10)	−0.0033 (11)	−0.0125 (11)
C13	0.0282 (13)	0.0291 (13)	0.0327 (14)	−0.0108 (11)	0.0017 (11)	−0.0109 (11)
C14	0.0326 (14)	0.0255 (13)	0.0263 (13)	−0.0049 (10)	−0.0059 (11)	−0.0019 (10)
C15	0.0272 (13)	0.0222 (12)	0.0291 (13)	−0.0042 (10)	−0.0043 (10)	−0.0064 (10)
C16	0.0325 (15)	0.0487 (17)	0.0395 (16)	−0.0112 (13)	0.0053 (12)	−0.0128 (13)
C17A	0.030 (4)	0.034 (4)	0.028 (3)	−0.004 (3)	−0.006 (3)	0.000 (3)
C18A	0.082 (7)	0.036 (5)	0.039 (4)	−0.009 (5)	−0.003 (4)	−0.012 (4)
C19A	0.031 (5)	0.041 (5)	0.035 (4)	0.001 (4)	−0.002 (4)	−0.013 (4)
C17B	0.030 (4)	0.041 (4)	0.033 (3)	−0.001 (3)	0.001 (3)	−0.002 (3)
C18B	0.039 (4)	0.047 (5)	0.038 (4)	−0.008 (3)	0.005 (3)	−0.016 (3)
C19B	0.041 (5)	0.062 (6)	0.045 (5)	0.006 (4)	0.015 (4)	−0.015 (4)
C20	0.0349 (14)	0.0268 (13)	0.0306 (13)	−0.0009 (11)	−0.0032 (11)	−0.0119 (10)
C21	0.0544 (18)	0.0287 (14)	0.0329 (14)	−0.0108 (13)	0.0035 (13)	−0.0061 (11)

### Geometric parameters (Å, °)

**Table:**

O1—C8	1.236 (3)	C15—H15A	0.9300
N1—C7	1.284 (3)	C16—C17A	1.502 (7)
N1—N2	1.379 (3)	C16—C17B	1.502 (7)
N2—C8	1.349 (3)	C16—H16A	0.9600
N2—H1N2	0.86 (2)	C16—H16B	0.9599
C1—C2	1.381 (3)	C16—H16C	0.9600
C1—C6	1.407 (3)	C16—H16D	0.9600
C1—H1A	0.9300	C17A—C19A	1.519 (13)
C2—C3	1.384 (3)	C17A—C18A	1.519 (14)
C2—H2A	0.9300	C17A—H17A	0.9800
C3—C4	1.387 (3)	C18A—H18A	0.9600
C3—H3A	0.9300	C18A—H18B	0.9600
C4—C5	1.386 (3)	C18A—H18C	0.9600
C4—H4A	0.9300	C19A—H19A	0.9600
C5—C6	1.387 (3)	C19A—H19B	0.9600
C5—H5A	0.9300	C19A—H19C	0.9600
C6—C7	1.491 (3)	C17B—C18B	1.511 (12)
C7—C20	1.506 (3)	C17B—C19B	1.533 (12)
C8—C9	1.524 (3)	C17B—H17B	0.9800
C9—C10	1.525 (3)	C18B—H18D	0.9600
C9—C21	1.529 (3)	C18B—H18E	0.9600
C9—H9A	0.9800	C18B—H18F	0.9600
C10—C15	1.385 (3)	C19B—H19D	0.9600
C10—C11	1.393 (3)	C19B—H19E	0.9600
C11—C12	1.386 (3)	C19B—H19F	0.9600
C11—H11A	0.9300	C20—H20A	0.9600
C12—C13	1.391 (3)	C20—H20B	0.9600
C12—H12A	0.9300	C20—H20C	0.9600
C13—C14	1.396 (3)	C21—H21A	0.9600
C13—C16	1.505 (3)	C21—H21B	0.9600
C14—C15	1.385 (3)	C21—H21C	0.9600
C14—H14A	0.9300		
			
C7—N1—N2	119.18 (19)	C17B—C16—C13	116.1 (3)
C8—N2—N1	119.6 (2)	C17A—C16—H16A	106.5
C8—N2—H1N2	116.6 (16)	C17B—C16—H16A	127.9
N1—N2—H1N2	123.7 (16)	C13—C16—H16A	108.0
C2—C1—C6	120.5 (2)	C17A—C16—H16B	109.7
C2—C1—H1A	119.7	C17B—C16—H16B	84.1
C6—C1—H1A	119.7	C13—C16—H16B	108.4
C1—C2—C3	120.7 (2)	H16A—C16—H16B	107.5
C1—C2—H2A	119.6	C17A—C16—H16C	79.8
C3—C2—H2A	119.6	C17B—C16—H16C	106.1
C2—C3—C4	119.4 (2)	C13—C16—H16C	108.5
C2—C3—H3A	120.3	H16B—C16—H16C	131.7
C4—C3—H3A	120.3	C17A—C16—H16D	129.3
C5—C4—C3	120.0 (2)	C17B—C16—H16D	109.7
C5—C4—H4A	120.0	C13—C16—H16D	108.5
C3—C4—H4A	120.0	H16A—C16—H16D	79.4
C4—C5—C6	121.4 (2)	H16C—C16—H16D	107.7
C4—C5—H5A	119.3	C16—C17A—C19A	111.3 (6)
C6—C5—H5A	119.3	C16—C17A—C18A	114.6 (8)
C5—C6—C1	117.9 (2)	C19A—C17A—C18A	111.4 (7)
C5—C6—C7	120.8 (2)	C16—C17A—H17A	106.3
C1—C6—C7	121.3 (2)	C19A—C17A—H17A	106.3
N1—C7—C6	115.09 (19)	C18A—C17A—H17A	106.3
N1—C7—C20	126.2 (2)	C16—C17B—C18B	114.0 (7)
C6—C7—C20	118.7 (2)	C16—C17B—C19B	111.2 (6)
O1—C8—N2	120.1 (2)	C18B—C17B—C19B	110.3 (7)
O1—C8—C9	121.5 (2)	C16—C17B—H17B	107.0
N2—C8—C9	118.3 (2)	C18B—C17B—H17B	107.0
C8—C9—C10	108.12 (19)	C19B—C17B—H17B	107.0
C8—C9—C21	110.8 (2)	C17B—C18B—H18D	109.5
C10—C9—C21	112.25 (19)	C17B—C18B—H18E	109.5
C8—C9—H9A	108.5	H18D—C18B—H18E	109.5
C10—C9—H9A	108.5	C17B—C18B—H18F	109.5
C21—C9—H9A	108.5	H18D—C18B—H18F	109.5
C15—C10—C11	117.8 (2)	H18E—C18B—H18F	109.5
C15—C10—C9	121.3 (2)	C17B—C19B—H19D	109.5
C11—C10—C9	120.9 (2)	C17B—C19B—H19E	109.5
C12—C11—C10	121.2 (2)	H19D—C19B—H19E	109.5
C12—C11—H11A	119.4	C17B—C19B—H19F	109.5
C10—C11—H11A	119.4	H19D—C19B—H19F	109.5
C11—C12—C13	121.2 (2)	H19E—C19B—H19F	109.5
C11—C12—H12A	119.4	C7—C20—H20A	109.5
C13—C12—H12A	119.4	C7—C20—H20B	109.5
C12—C13—C14	117.3 (2)	H20A—C20—H20B	109.5
C12—C13—C16	121.5 (2)	C7—C20—H20C	109.5
C14—C13—C16	121.3 (2)	H20A—C20—H20C	109.5
C15—C14—C13	121.5 (2)	H20B—C20—H20C	109.5
C15—C14—H14A	119.3	C9—C21—H21A	109.5
C13—C14—H14A	119.3	C9—C21—H21B	109.5
C14—C15—C10	121.0 (2)	H21A—C21—H21B	109.5
C14—C15—H15A	119.5	C9—C21—H21C	109.5
C10—C15—H15A	119.5	H21A—C21—H21C	109.5
C17A—C16—C13	116.4 (3)	H21B—C21—H21C	109.5
			
C7—N1—N2—C8	173.9 (2)	C21—C9—C10—C11	50.4 (3)
C6—C1—C2—C3	0.4 (3)	C15—C10—C11—C12	2.5 (3)
C1—C2—C3—C4	−0.6 (4)	C9—C10—C11—C12	−176.96 (19)
C2—C3—C4—C5	0.4 (4)	C10—C11—C12—C13	−1.6 (3)
C3—C4—C5—C6	0.1 (4)	C11—C12—C13—C14	−0.2 (3)
C4—C5—C6—C1	−0.4 (3)	C11—C12—C13—C16	179.1 (2)
C4—C5—C6—C7	179.1 (2)	C12—C13—C14—C15	1.0 (3)
C2—C1—C6—C5	0.1 (3)	C16—C13—C14—C15	−178.3 (2)
C2—C1—C6—C7	−179.3 (2)	C13—C14—C15—C10	−0.1 (3)
N2—N1—C7—C6	−179.98 (19)	C11—C10—C15—C14	−1.7 (3)
N2—N1—C7—C20	−1.1 (4)	C9—C10—C15—C14	177.78 (19)
C5—C6—C7—N1	167.0 (2)	C12—C13—C16—C17A	−113.3 (6)
C1—C6—C7—N1	−13.6 (3)	C14—C13—C16—C17A	66.0 (6)
C5—C6—C7—C20	−12.0 (3)	C12—C13—C16—C17B	−81.6 (6)
C1—C6—C7—C20	167.4 (2)	C14—C13—C16—C17B	97.7 (5)
N1—N2—C8—O1	179.2 (2)	C17B—C16—C17A—C19A	80.4 (10)
N1—N2—C8—C9	−3.9 (4)	C13—C16—C17A—C19A	176.9 (5)
O1—C8—C9—C10	92.1 (3)	C17B—C16—C17A—C18A	−47.1 (11)
N2—C8—C9—C10	−84.7 (3)	C13—C16—C17A—C18A	49.4 (12)
O1—C8—C9—C21	−31.3 (4)	C17A—C16—C17B—C18B	42.9 (10)
N2—C8—C9—C21	151.9 (2)	C13—C16—C17B—C18B	−55.0 (10)
C8—C9—C10—C15	108.5 (2)	C17A—C16—C17B—C19B	−82.6 (10)
C21—C9—C10—C15	−129.0 (2)	C13—C16—C17B—C19B	179.6 (4)
C8—C9—C10—C11	−72.0 (3)		

### Hydrogen-bond geometry (Å, °)

**Table:**

*D*—H···*A*	*D*—H	H···*A*	*D*···*A*	*D*—H···*A*
N2—H1N2···O1^i^	0.86 (2)	2.08 (2)	2.928 (3)	173 (2)
C20—H20A···O1^i^	0.96	2.31	3.247 (3)	165
C20—H20B···Cg1^ii^	0.96	2.75	3.609 (3)	150

Symmetry codes: (i) −*x*, −*y*+2, −*z*; (ii) *x*−1, *y*, *z*.
